# Intestinal organoid-based 2D monolayers mimic physiological and pathophysiological properties of the pig intestine

**DOI:** 10.1371/journal.pone.0256143

**Published:** 2021-08-23

**Authors:** Pascal Hoffmann, Nadine Schnepel, Marion Langeheine, Katrin Künnemann, Guntram A. Grassl, Ralph Brehm, Bettina Seeger, Gemma Mazzuoli-Weber, Gerhard Breves

**Affiliations:** 1 Institute for Physiology and Cell Biology, University of Veterinary Medicine Hannover, Hannover, Germany; 2 Institute for Anatomy, University of Veterinary Medicine Hannover, Hannover, Germany; 3 Institute of Medical Microbiology and Hospital Epidemiology and German Center for Infection Research (DZIF), Partner Site Hannover, Hannover Medical School, Hannover, Germany; 4 Institute for Food Quality and Food Safety, University of Veterinary Medicine Hannover, Hannover, Germany; Texas A&M University, UNITED STATES

## Abstract

Gastrointestinal infectious diseases remain an important issue for human and animal health. Investigations on gastrointestinal infectious diseases are classically performed in laboratory animals leading to the problem that species-specific models are scarcely available, especially when it comes to farm animals. The 3R principles of Russel and Burch were achieved using intestinal organoids of porcine jejunum. These organoids seem to be a promising tool to generate species-specific *in vitro* models of intestinal epithelium. 3D Organoids were grown in an extracellular matrix and characterized by qPCR. Organoids were also seeded on permeable filter supports in order to generate 2D epithelial monolayers. The organoid-based 2D monolayers were characterized morphologically and were investigated regarding their potential to study physiological transport properties and pathophysiological processes. They showed a monolayer structure containing different cell types. Moreover, their functional activity was demonstrated by their increasing transepithelial electrical resistance over 18 days and by an active glucose transport and chloride secretion. Furthermore, the organoid-based 2D monolayers were also confronted with cholera toxin derived from *Vibrio cholerae* as a proof of concept. Incubation with cholera toxin led to an increase of short-circuit current indicating an enhanced epithelial chloride secretion, which is a typical characteristic of cholera infections. Taken this together, our model allows the investigation of physiological and pathophysiological mechanisms focusing on the small intestine of pigs. This is in line with the 3R principle and allows the reduction of classical animal experiments.

## Introduction

Until today laboratory animals are frequently used to investigate physiological and pathophysiological functions of the gastrointestinal tract. This includes studies on the interaction of the intestinal epithelium with pathogens and their enterotoxins. An approach for investigating such topics using alternative models is the usage of cell culture-based systems. However, when such experiments focus on livestock, such as pigs, which are not only used for studying pig-specific diseases but also extensively as model for human intestinal pathophysiology [reviewed by 1], alternative models are still limited. Porcine cell lines, such as the small intestine-derived IPEC-J2 cells [2] and the colon-derived cell line PoCo83-3 [3] serve as a potential approach to investigate pathogen-host interactions [4, 5]. However, these cell lines only contain enterocytes and lack other cell types such as goblet cells. The organoid technique was introduced in 2009 [6]; this method allows displaying the complete cellular composition of the intestinal epithelium, providing a better model to compare with the *in vivo* situation. Since then, porcine organoids from juvenile [7] and from adult pigs [8] have been successfully cultured. In addition, organoids can be dissociated and grown in a 2D-monolayer system [9] and this approach has been proposed as an optimal tool for drug discovery and drug development as reviewed by Olayanju, Jones [10]. However, despite the application of this tool in many fields, relevant physiological transport characteristics as well as pathophysiological reactions to enterotoxins within this system have not been characterized. Therefore, the initial aim of our study was to establish a porcine intestinal organoid-based system to investigate the physiological transport properties of the intestinal epithelium using the Ussing chamber method. This could be compared with and eventually replace the use of classical animal-derived tissues. After characterizing this model our second aim was to prove its suitability for studying pathophysiological mechanisms: thus, we applied cholera toxin (CTX) in this system and investigated its pathophysiological effects.

## Materials & methods

### Generation of intestinal organoids

Intestinal organoids were generated from intestinal crypts [11]. One healthy pig (61.5 kg) was sacrificed by captive bolt shoot and bleeding. According to the Animal Protection Law, this (slaughter and removal of tissues) is not classified as animal experiment but has to be announced to the university’s animal welfare officer (registration no. TiHo-T-2017-22). The intestinal crypts were obtained by dissecting a 10 cm long part of the porcine jejunum, which was flushed with ice cold PBS, opened lengthwise with scissors, cut into 2–4 cm pieces and washed three times with ice cold PBS in a conical tube. The supernatant was discarded after each washing step. Pieces of the intestine were further processed into pieces with a size smaller than 0.5 cm, transferred to a tube prefilled with 10 ml ice cold crypt chelating buffer (0.01 M ethylenediamine tetraacetic acid (EDTA) in PBS, pH 8 [Sigma- Aldrich, Schnelldorf, Germany]) and placed on ice on an orbital shaker on gentle settings for 90 min. Afterwards, the intestinal fragments were allowed to settle at the bottom of the tube and the supernatant was discarded. Five ml of ice-cold PBS were added and pipetted up and down 20 times with a glass pipet. Fragments were allowed to settle at the bottom of the tube and the supernatant transferred to a fresh tube. This process was repeated two times and the supernatant was centrifuged 5 min, 200 x *g* and 4°C. The pellet was resuspended in ice cold PBS and the crypts were counted. The solution was centrifuged again as described above and 25 μl culture medium (S1 Table) per 1,000 crypts was used to resolve the pellet. Furthermore, 25 μl Matrigel (FALC354234, Omnilab, Bremen, Germany) per 1,000 crypts was added. Crypt suspension at a volume of 50 μl each was applied to a pre-warmed 24-well plate and cultured as described below.

### Cultivation of 3D organoids

3D organoids were cultured at 37°C and 5% CO_2_, with the culture medium being changed every two to three days. For weekly subcultivation of the organoids, culture medium was removed and 1 ml ice cold PBS added to each well. Matrigel was broken by pipetting the PBS 20 times with a 1,000 μl tip and another 15 times with a 200 μl tip mounted on a 1,000 μl tip. Organoids were aspirated and transferred to a pre-cooled tube. Four wells of 3D organoids were pooled at this stage. Organoids were centrifuged at 200 x *g*, 5 min at 4°C. Supernatant was discarded and the pellet resuspended in 50 μl culture medium and mixed with 200 μl Matrigel. 50 μl of this combination was transferred to a fresh well of a pre-heated (37°C) 24-well plate. Matrigel was allowed to solidify in the incubator at 37°C for 45 min and was overlaid with 500 μl culture medium per well.

### RNA isolation, reverse transcription and quantitative real-time PCR

At every weekly subcultivation, organoids were collected in a tube pre-filled with 10 ml ice-cold PBS and centrifuged for 10 min at 4°C and 200 x *g*. Supernatant was discarded. Pellet was resuspended in 1 ml PBS and transferred to a new tube. After centrifugation at 16,100 x *g* at 4°C for 10 minutes, supernatant was discarded and the organoids stored at -80°C until further processing. RNA extraction, reverse transcription and qPCR analysis were conducted as described before [12]. Primers of genes of physiological interest for this work are listed in S2 Table.

### Organoid-based 2D monolayer culture

Generation of an organoid-based 2D monolayer culture was performed by removing supernatant from wells containing 3D organoids and subsequent addition of 1 ml ice cold PBS. Matrigel was dissected by pipetting and organoids were collected in a tube pre-filled with 10 ml ice cold PBS. After centrifugation at 4°C and 600 x *g* for 10 min, supernatant was discarded. Pellet was resuspended in 0.05% Trypsin/EDTA and incubated for 5 min at 37°C before resuspending the solution 20 times with a 1,000 μl tip and another 15 times with a 200 μl tip mounted on a 1,000 μl tip. 10% (v/v) ice cold fetal bovine serum in DMEM was added and the tube was centrifuged at 3,000 x *g*, 4°C for 10 min. Supernatant was discarded and the pellet resuspended in monolayer medium (S3 Table). Cells were counted and 2 * 10^5^ cells seeded on precoated (Matrigel 1:40 in PBS) Snapwells^®^ (Corning, Kaiserslautern, Germany; diameter: 12 mm; pore size: 0.4 μm). Basolateral chamber was filled with 3 ml monolayer medium, apical chamber with 0.5 ml monolayer medium, respectively. After 16 days of cultivation, medium was changed to differentiation medium for another 2 days (S4 Table) until experiments were conducted after a total incubation time of 18 days.

### Transepithelial electrical resistance measurements

To determine cell monolayer integrity, transepithelial electrical resistance (TEER) was measured using an epithelial volt-ohm-meter (EVOM^2^; WPI, Berlin, Germany). Measurements were performed simultaneously to each culture medium exchange of organoids cultured on Snapwells^®^ by measuring the TEER of wells with organoids corrected by the respective value of wells containing no organoids (blank) according to the manufacturer.

### Ussing chamber experiments—transport physiology

Organoids cultured on Snapwells^®^ were mounted in Ussing cambers [13], mimicking the mucosal and the serosal side of the intestine. Ussing chambers were connected to a computer-controlled voltage clamp (K. Mussler, Aachen, Germany). Each compartment was filled with 5 ml of the respective buffers (S5 Table), which were heated to 37°C and aerated with carbogen. After an equilibration phase of 5 min, the tissues were set to short-circuit conditions at 0 mV to impede electrogenic transport.

#### Ussing chamber—cellular characterization

Fifteen min after setting the short-circuit conditions to 0 mV, organoid-based 2D monolayers were incubated with glucose (10 mM, mucosal, diluted in *aqua destillata*) and mannitol (10 mM, serosal for osmolality equilibration, diluted in *aqua destillata*) for 20 min, followed by a 15 min incubation with forskolin (10 μM, serosal, Sigma-Aldrich, diluted in Dimethyl sulfoxide) and with carbachol (10 μM, serosal, Sigma-Aldrich, diluted in *aqua destillata*) for another 10 min. Indometacin (10 μM, diluted in *aqua destillata*) was added to impede prostaglandin synthesis and to avoid spontaneous chloride (Cl^-^) secretion [14]. Throughout the experiment, both, short-circuit currents (I_sc_) and resistances (R_t_) were measured. At each experimental run three chambers were performed as technical replicates.

#### Ussing chamber—incubation with bacteria-derived toxins

Fifteen min after the tissues were set to short-circuit conditions, organoid-based 2D monolayers were incubated for 80 min with 7.5 μg/ml CTX (CAS 9012-63-9; Enzo Life Sciences, Lörrach, Germany, diluted in *aqua destillata*). This was followed by the addition of 10 μM forskolin to the serosal chamber for 15 min and a final incubation of 10 min with 100 μM ouabain (Sigma-Aldrich) on the serosal side. I_sc_ and R_t_ were measured continuously. Technical replicates were obtained as mentioned above.

### Histological analysis

#### Analysis of mucus layer formation

Mucus layer formation was analyzed by mucin staining with periodic acid-Schiff reagent (PAS). Organoids were cultivated on Snapwells^®^ as described above, before the Snapwell^®^ membranes were cut out, fixed in Bouin solution for 10 min and then rinsed three times with PBS. After fixation, the membranes were cut in two pieces and embedded in 5% (w/v) agarose followed by embedding in paraffin. For morphological evaluation, 3 μm-slices were sectioned and hematoxylin and eosin (H & E) staining was performed. In order to confirm the existence of goblet cells, de-paraffinized slides were stained with PAS, dehydrated and mounted with Eukitt^®^ (O. Kindler GmbH, Freiburg, Germany), all according to standard protocols [15].

#### Immunohistochemical staining

Immunohistochemical staining of zonula occludens-1 protein (ZO-1) and cystic fibrosis transmembrane conductance regulator (CFTR) was obtained by fixation, embedding and sectioning of organoids on Snapwell^®^ membranes as described above. After de-paraffinization and inhibition of endogenous peroxidase activity with 3% H_2_O_2_ in 80% ethanol for 30 min, ZO-1, Claudin-2 and Claudin-3 sections were microwave-pretreated with sodium citrate buffer (pH 6.0) for 3 x 5 min at 800 Watts and allowed to cool down to room temperature for 30 min. CFTR sections were heated to 96–99°C in tris-EDTA-citrate buffer (pH 7,8) for 20 min, allowed to cool down to 60°C and washed 5 min in TBST [15]. Afterwards, slides for both stainings were blocked with 3% BSA for 20 min, before they were incubated with the primary antibody (ZO-1: Santa Cruz Biotechnology, Dallas, USA; catalog no.: sc-10804, dilution factor: 1:80; Claudin-2: Biologo, Kronshagen, Germany; catalog no.: CLA002, dilution factor 1:50; Claudin-3, Invitrogen, Waltham, USA: catalog no.; 34–1700, dilution factor 1:1,000; CFTR: Cell Signaling Technology, Danvers, USA; catalog no.: 78335, dilution factor: 1:100) overnight at 4°C. The sections were then exposed to the secondary antibody (biotin-labelled goat-anti-rabbit; Vector, Burlingame, USA; catalog no.: BA-1000; dilution factor: 1:200) for 60 min at room temperature and, after rinsing, 30 min with the ABC-System (Vector; catalog no.: PK-6100) according to the manufacturer’s protocol. After visualization with 3,3′-Diaminobenzidine (DAB), the sections were briefly counterstained with hematoxylin for 10 sec and rinsed with running water. Finally, the slides were dehydrated and mounted with Eukitt®. Negative and isotype controls were included in the analyses and images were captured using a Zeiss Axioskop (Zeiss, Jena, Germany) with an Olympus SC50 camera controlled by the Olympus CellSens software (Olympus Soft Imaging Solutions GmbH, Germany).

#### Immunofluorescence staining

Immunofluorescence was performed by cultivating organoid-based 2D monolayers on Snapwells® as described above. Fixation, embedding and sectioning of organoids on Snapwell^®^ membranes were carried out as described above. After de- paraffinization, monolayers used for Villin staining were heated to 96–99°C in tris-EDTA-citrate buffer (pH 7.8) for 20 min, allowed to cool down to 60°C and washed 5 min in TBST [15]. All sections were permeabilized for 60 min using 0.25% Triton X-100/PBS and blocked with 5% goat-serum in PBST for one hour. Primary anti-e-cadherin (Abcam, Cambridge, UK; catalog no.: ab76055, dilution factor 1:500), anti-villin (Abcam; catalog no.: ab130751, dilution factor 1:250) and anti-chromogranin A (Immunostar, Hudson, USA; catalog no.: 20085, dilution factor: 1:1,000) were incubated with the slides at 4°C overnight. After washing thrice with PBS, incubation with the secondary fluorescence-labelled antibody (donkey anti-mouse for e-cadherin (Invitrogen; catalog no.: A10036; dilution factor: 1:1,000) and goat-anti-rabbit (Sigma-Aldrich; catalog no.: SAB4600084; dilution factor: 1:1,000) for villin and chromogranin A) was performed for 60 min at room temperature. Counterstaining of nuclei was done via DAPI staining and cover glasses were embedded using Pro Long Gold (Invitrogen, USA). Analysis was performed using a Zeiss Axiovert 200M microscope attached to a Zeiss AxioVision Imaging System. Numeric apertures of the objective lens used was: 40 x oil/NA 1.3.

### Data analysis and statistics

Basal values of I_sc_ and R_t_ were determined by calculating the arithmetic mean of the last 10 data points before the addition of an agent. Changes in short-circuit currents (ΔI_sc_) were calculated by subtracting the arithmetic mean from the maximal value after the addition of each agent. The normal distribution of the residuals was tested using the D’Agostino & Pearson test. A paired *t*-test was used to compare datasets. All statistical analyses were performed using Prism version 8.0.1 (GraphPad, San Diego, USA) and *p* values ≤ 0.05 were considered statistically significant.

## Results

### 3D-organoids developed from intestinal crypts

**Fig 1:**
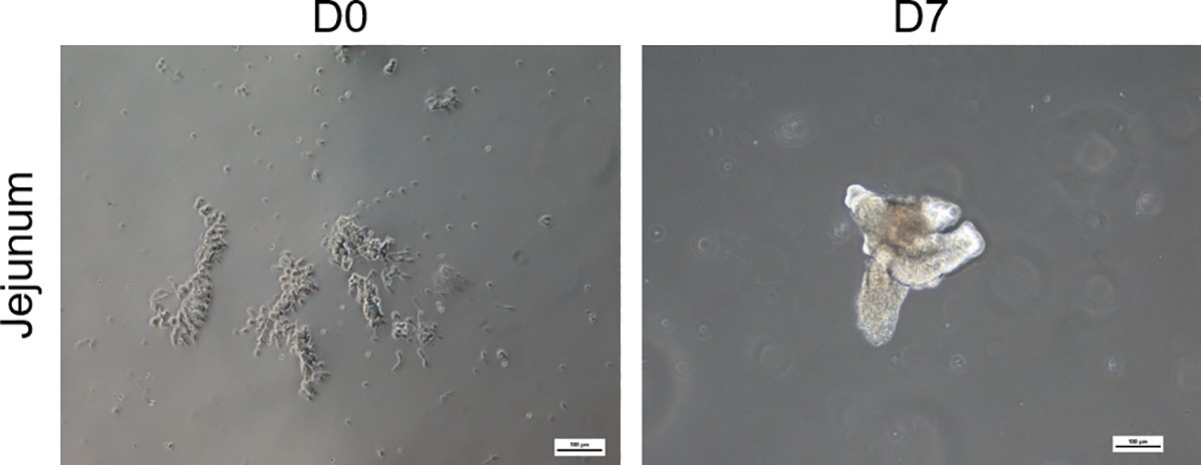
Intestinal porcine crypts isolated from the jejunum (D0) form 3D-organoids (D7) after a cultivation period of seven days.

Isolated intestinal crypts of the porcine jejunum embedded in Matrigel^®^ formed 3D-organoid structures during a cultivation period of seven days (Fig 1).

### Gene expression of porcine 3D organoids

**Fig 2:**
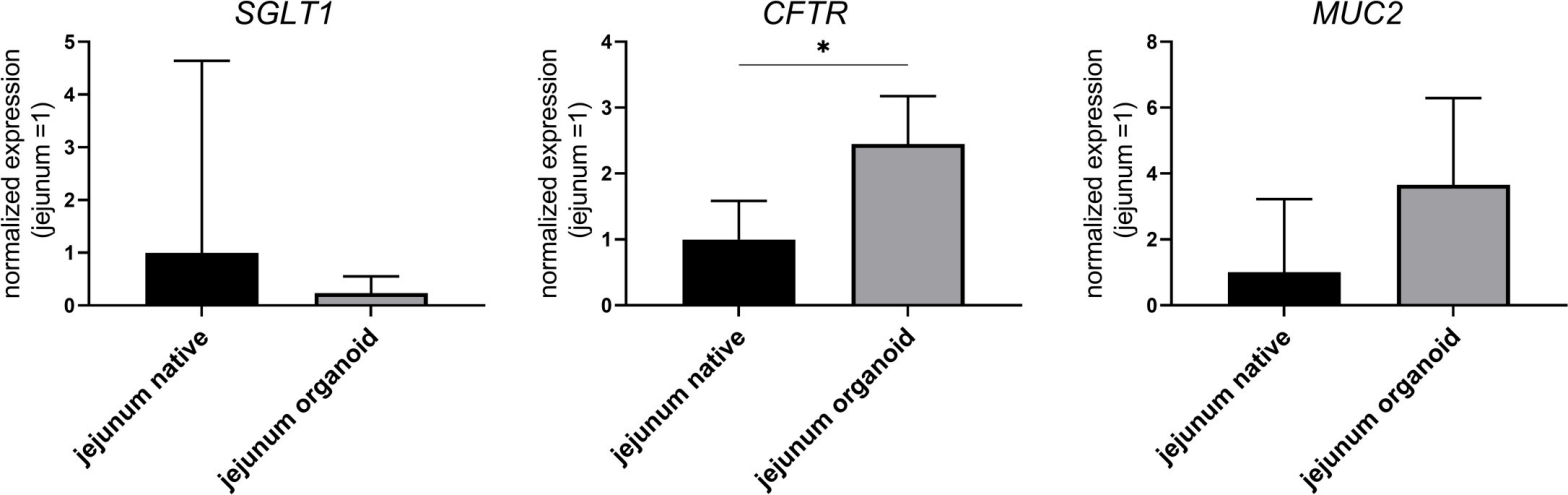
Gene expression of sodium/glucose cotransporter 1 (*SGLT1*), cystic fibrosis transmembrane conductance regulator (*CFTR*) and mucin 2 (*MUC2*) in intestinal organoids of the porcine jejunum cultivated for seven days in comparison to native tissue. Reference genes ribosomal protein S23 and ribosomal protein P0 were used for normalization of expression. Values shown are the geometric mean ± geometric standard deviation (SD) of four independent experiments. * p < 0.05.

Gene expression of sodium/glucose cotransporter 1 (*SGLT1*), cystic fibrosis conductance transmembrane conductance regulator (*CFTR*) and mucin 2 (*MUC2*) was determined in 3D organoids and compared with gene expression in native porcine jejunum. While the expression of *SGLT1* as well as *MUC2* showed no differences between organoids and native epithelium, *CFTR* expression was significantly higher in organoids (Fig 2).

### Cellular monolayer and mucin production

**Fig 3:**
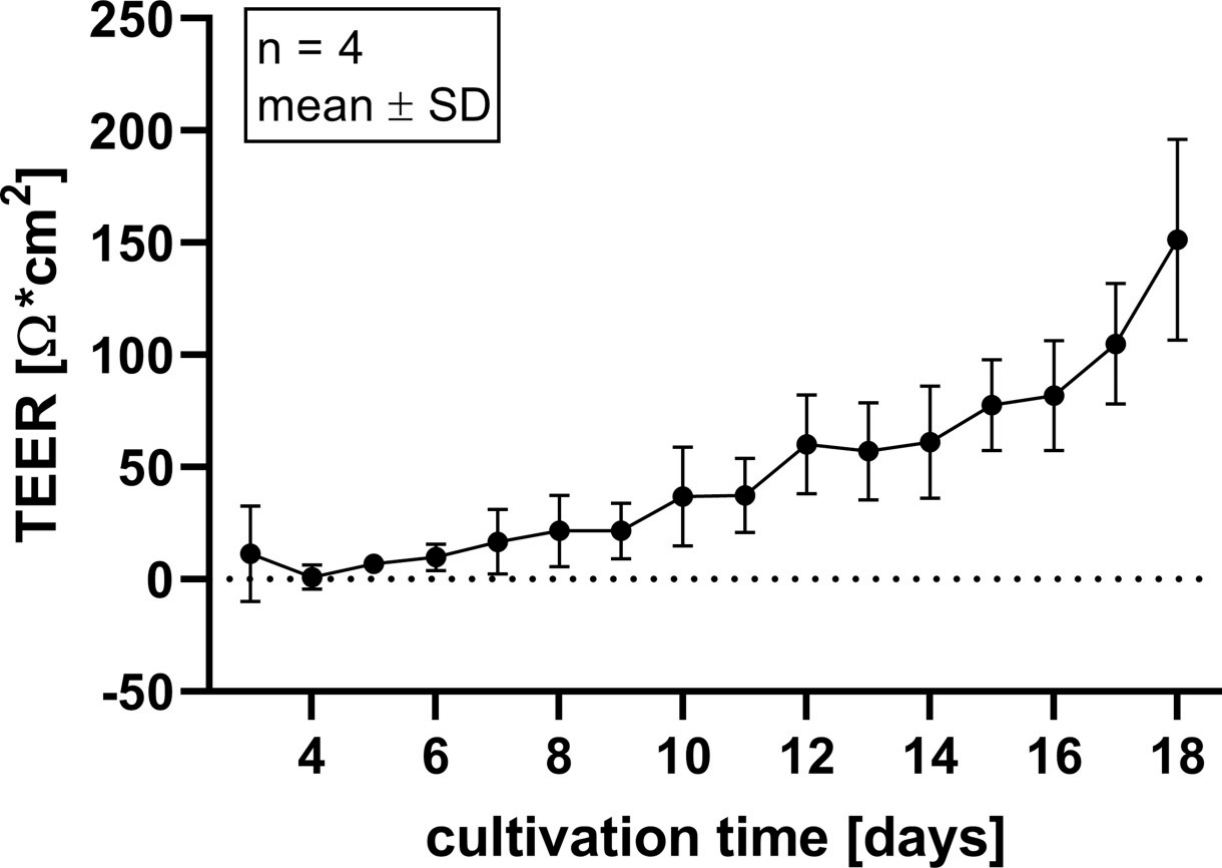
Changes of transepithelial electrical resistance (TEER) values over the 18 day culture period on Snapwells^®^ of organoid-based 2D monolayers as a function of time. Values shown are the mean ± SD of four independent experiments (passages).

**Fig 4:**
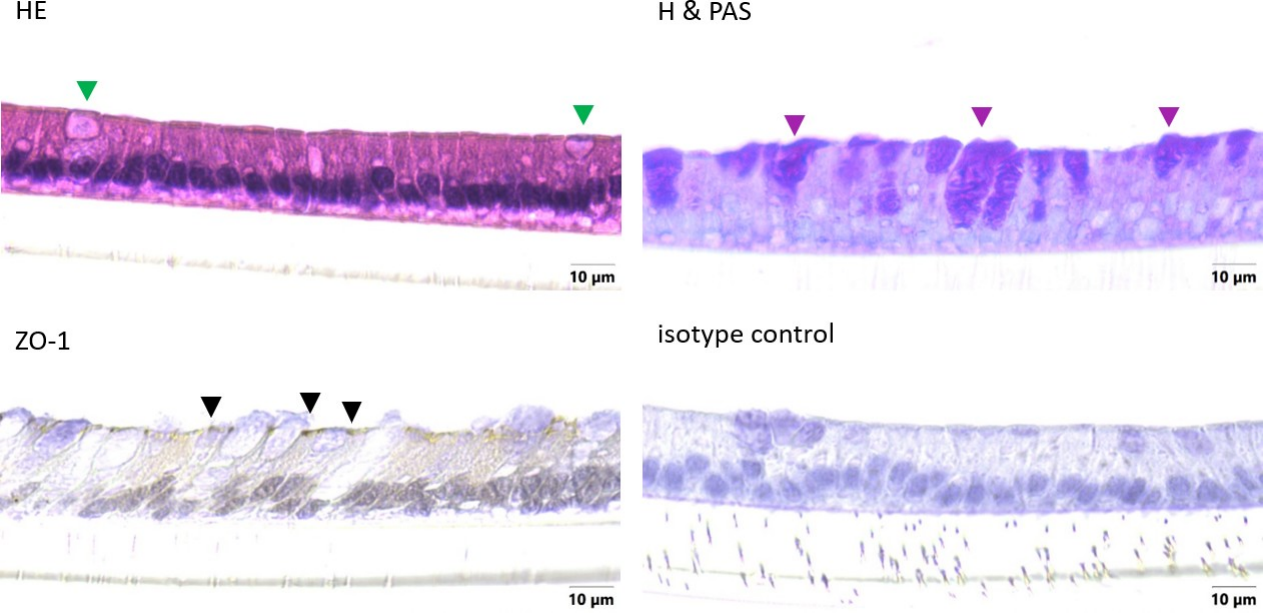
Cellular arrangement (cross sections) of jejunum organoids grown in 2D on Snapwells^®^ for 18 days. Cells were either stained with hematoxylin and eosin (H & E) or hematoxylin and PAS (H & PAS) as well as immunohistochemical staining (including isotype controls) of ZO-1 (cross-section). Green triangles indicate cells with a goblet, cup-like appearance, violet triangles indicate PAS-positive cells and black triangles show a positive signal of ZO-1. Representative figures are shown, chosen from three independent experiments with three technical replicates per staining.

**Fig 5:**
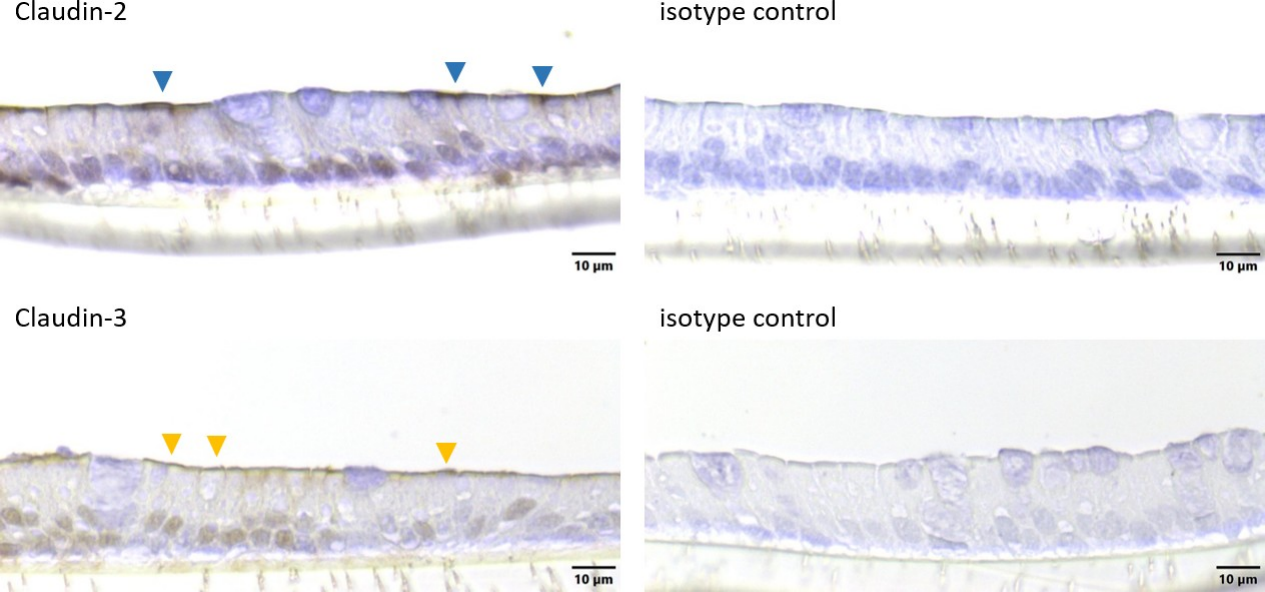
Cellular arrangement (cross sections) of jejunum organoids grown in 2D on Snapwells^®^ for 18 days. Immunohistochemical staining (including isotype controls) of Claudin-2 and Claudin-3 was performed. Blue triangles indicate ceklls with positive Claudin-2 signal, orange triangles indicate cells with a positive Claudin-3 signal. Representative figures are shown, chosen from three independent experiments with three technical replicates per staining.

**Fig 6:**
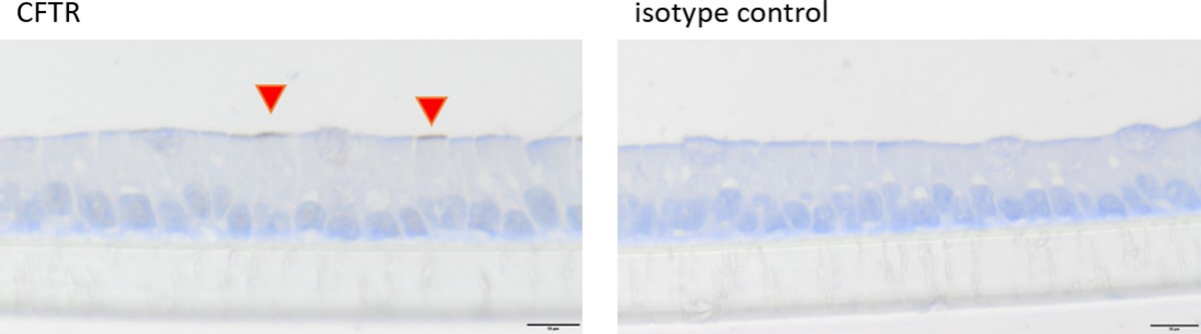
Cellular arrangement (cross sections) of jejunum organoids grown in 2D on Snapwells^®^ for 18 days. Immunohistochemical staining (including isotype controls) of CFTR was performed. Red triangles indicate cells with a positive signal of CFTR. Representative figures are shown, chosen from three independent experiments with three technical replicates per staining.

**Fig 7:**
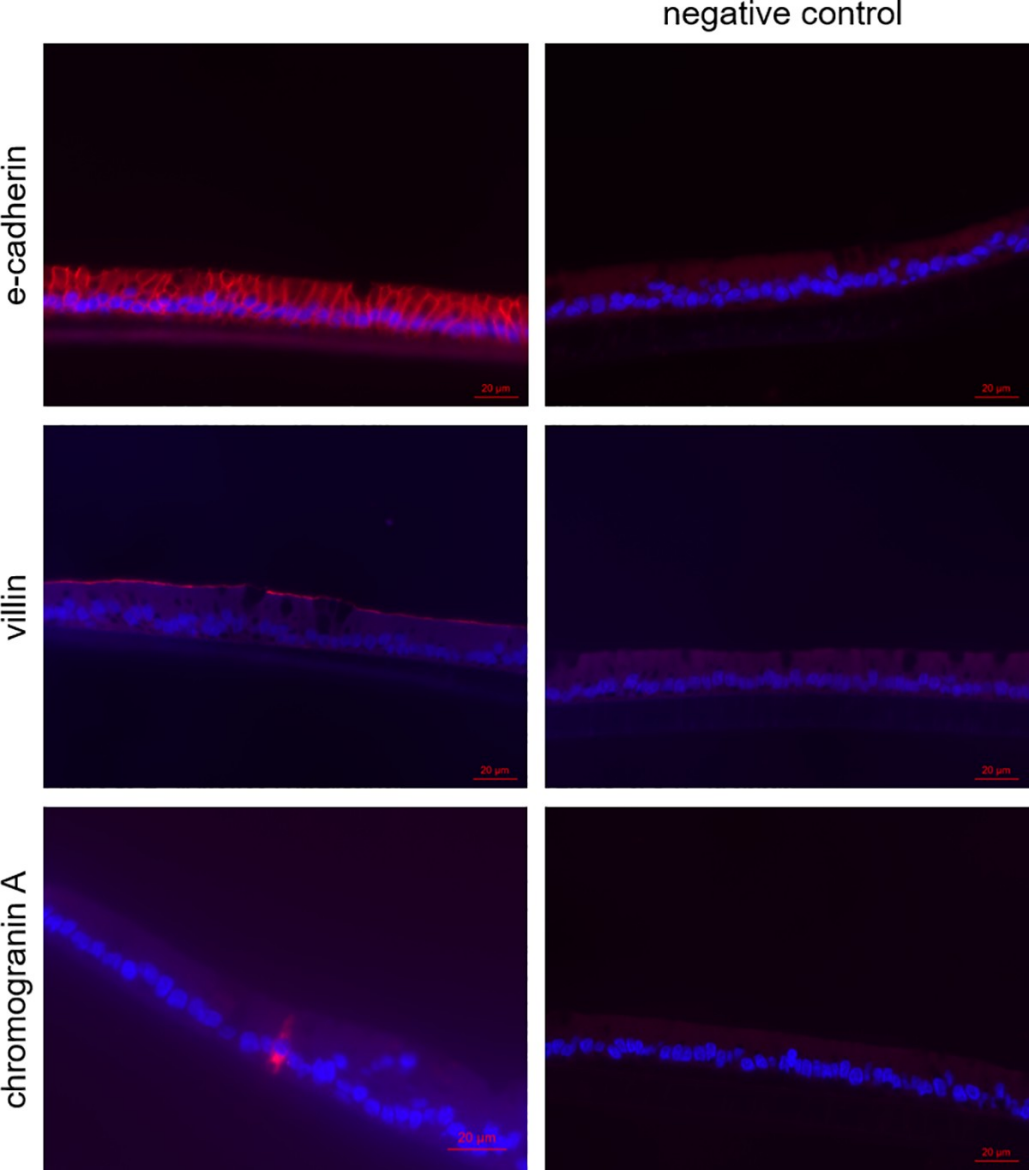
Immunofluorescence staining (cross sections) of jejunum organoids grown in 2D on Snapwells^®^ for 18 days. E-cadherin, villin and chromogranin A are displayed in red, nuclei are counterstained in blue. Representative figures are shown, chosen from three independent experiments with three technical replicates per cellular approach.

The integrity of the cellular layer was determined every day after medium change by measuring the TEER. After 18 days of cultivation, an electrical resistance of 151.4 ± 38.8 Ω*cm^2^ was reached (Fig 3). Organoids grown in a 2D manner on Snapwell^®^ membranes were stained with H & E as well as hematoxylin and PAS to determine cellular arrangement and the formation of mucus. A cellular monolayer could be detected including some cells with a goblet, cup-like appearance (green triangles, Fig 4). Hematoxylin and PAS staining showed a strong signal for some cells (violet triangles, Fig 4). Zonula occludens-1 staining showed a positive signal on the apical side of the cellular layer (black triangles, Fig 4), while the isotype control was negative. Immunohistochemical staining of Claudin-2 (blue triangles, Fig 5) and Claudin-3 (orange triangles, Fig 5) both showed a positive signal on the apical side of the cellular layer, while the isotype control was negative. Immunohistological staining of CFTR showed a strong signal on the apical membrane of some cells (red triangles, Fig 6). Immunofluorescence staining showed a positive signal for e-cadherin between the individual cells, villin signals were detected at the apical membrane of the cells while chromogranin A was only abundant in a few cells (Fig 7).

### Ussing chamber studies—transport characteristics

**Fig 8:**
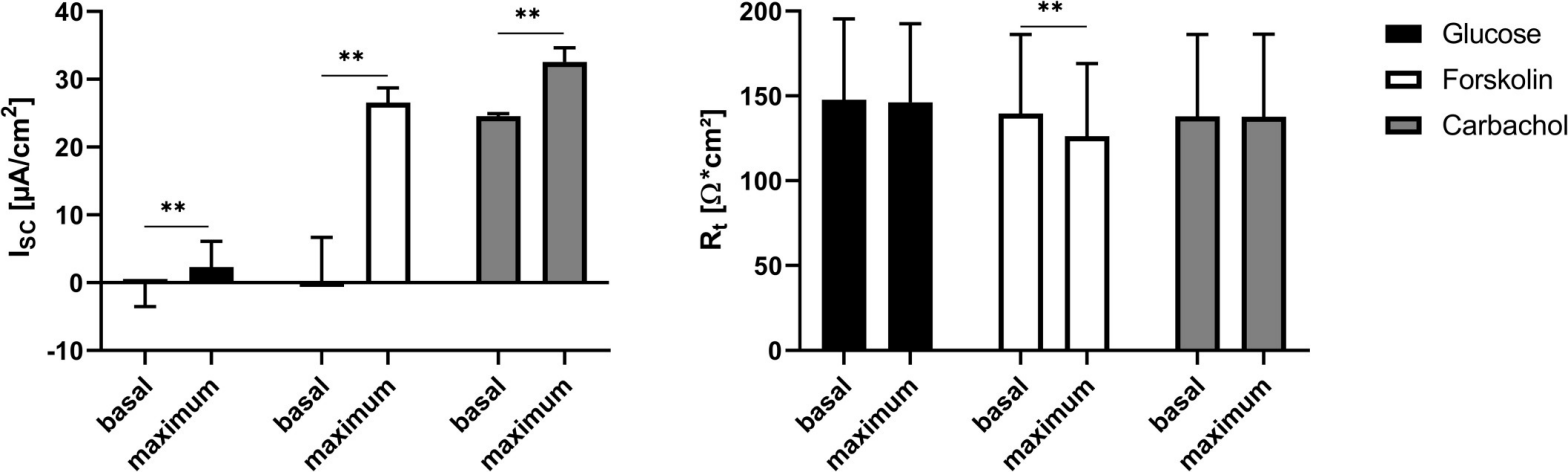
Basal and maximal I_sc_ and R_t_ values of organoid-based 2D monolayers cultivated for 18 days determined before and after subsequent addition of glucose, carbachol and forskolin. Values shown are the mean ± SD of four independent experiments. ** p < 0.01.

Physiological transport characteristics were investigated using the Ussing chamber system. Mucosal addition of 10 mM glucose led to a significant increase of the I_sc_. This was followed by the serosal incubation with 10 μM forskolin leading to an increase in I_sc_ as well as a decrease in R_t_. Final serosal addition of 10 μM carbachol increased the I_sc_ significantly (Fig 8).

### Ussing chamber studies—effects of CTX

**Fig 9:**
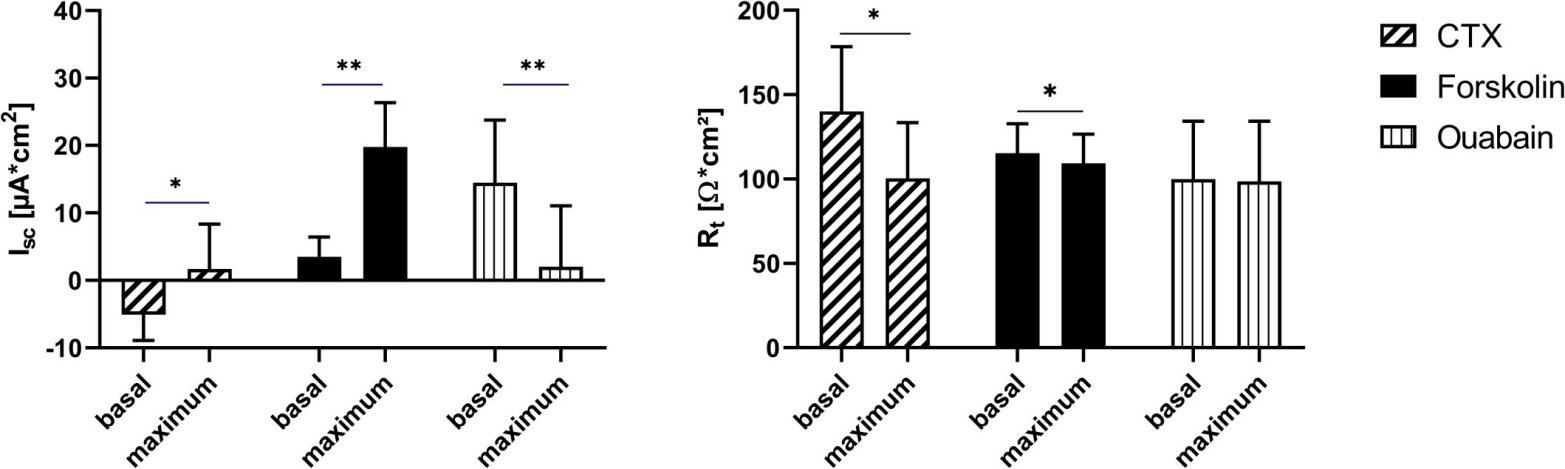
Basal and maximal I_sc_ and R_t_ values of organoid-based 2D monolayers cultivated for 18 days determined before and after subsequent addition of CTX, forskolin and ouabain. Values shown are the mean ± SD of four independent experiments. * p < 0.05; ** p < 0.01.

Incubating the organoid-based 2D monolayers with 7.5 μg/ml CTX from the mucosal side lead to a significant increase in I_sc_, while R_t_ decreased. Following serosal incubation with 10 μM forskolin also showed a significant increase of the I_sc_, while the R_t_ was decreased in a significant manner. Final serosal addition of 100 μM ouabain to the serosal chamber decreased in the I_sc_ significantly (Fig 9).

## Discussion

### Organoids are characterized by increasing electrical resistance as well as expression of goblet cells, enterocytes and tight junction associated proteins

Isolated crypts of the porcine jejunum generated 3D-organoids after a cultivation time of 7 days. This is mediated by the multipotent or so-called adult stem cells present in the intestinal crypts differentiating into the epithelial cells of the gut [16–18]. These organoids were further used to generate 2D monolayers. These organoids seeded in a 2D manner on Snapwells^®^ showed increasing TEER values up to about 150 Ω*cm^2^ after 18 days of cultivation compared to native porcine epithelium with approximately 120 Ω*cm^2^ [19]. The data obtained in this study contrast with data of van der Hee, Madsen [20], who showed an increase to 750–850 Ω*cm^2^. This discrepancy could be due to the slightly different cultivation conditions such as a varying medium composition. Histological analysis of our organoids revealed an intact monolayer including the expression of the tight junction-associated protein ZO-1, which is highly comparable to Caco-2/HT29-MTX based monolayers [21, 22] [Hoffmann et al. 2021, under revision at: PLOS ONE]. Furthermore, the adherens junction protein e-cadherin as well as the tight junction proteins Claudin-2 and Claudin-3 were shown to be expressed in organoid-based monolayers. Investigations in piglets showed developmental changes in intercellular junctions such as an increase in protein expression of Claudin-1, Claudin-3, occludin and ZO-1 during the suckling period [23]. This increase in tight junction protein expression may also be assumed during the cultivation process of organoids but needs to be demonstrated with further experiments. In addition to the expression of ZO-1, histochemical staining of organoid-based 2D monolayers showed the abundance of mucus-filled cells throughout the monolayer, which could further be supported by *MUC2* gene expression in the 3D organoids, therefore these cells can be identified as goblet cells. The amount of goblet cells detectable by immunohistochemistry in the monolayer appears to be slightly higher compared to native porcine jejunum, which only shows a moderate number of goblet cells [24]. This finding can be explained by the composition of the differentiation medium containing the gamma-secretase inhibitor DAPT, leading to increased formation of goblet cells [25]. Expression of villin shows mature enterocytes indicating the formation of the typical intestinal brush boarder membrane [9, 26]. Chromogranin A allowed the identification of single enteroendocrine cells in our organoid-based 2D model [9, 26]. The cellular composition of porcine 3D organoids has been shown in previous studies also indicating the presence of absorptive enterocytes, goblet cells and enteroendocrine cells [26, 27], but to our knowledge not in the 2D model under these cultivation conditions. The abundance of enterocytes, goblet cells and enteroendocrine cells provides a secretory, functionally active epithelium including a mucin-rich environment that serves as a barrier against infections and allows these organoids to serve as an optimal system for the underlying question.

Development of an intact cellular layer during cultivation as well as a cellular composition including absorptive enterocytes and goblet cells enables our model to be compared with native epithelium.

### Cellular characterization shows functional transport characteristics

Intestinal glucose transport is mainly mediated by the sodium/glucose cotransporter 1 (SGLT1) [28], resulting in an increase in I_sc_ in response to mucosal addition of glucose as shown in porcine organoids seeded on Snapwell^®^ inserts as well as in native porcine jejunum in earlier studies [29]. Abundance of SGLT1 and other transport proteins was shown in earlier studies on murine organoids [30, 31] and could also be confirmed in our study based on *SGLT1* gene expression. However, gene expression in organoids was lower compared to native epithelium which might explains the moderate increase in I_sc_ in response to mucosal addition of glucose. One reason could be the relatively high abundance of goblet cells in this model and the resulting low number of enterocytes which solely express *SGLT1* as already shown in rats [32].

Secretion of Cl^-^ by the cystic fibrosis transmembrane conductance regulator (CFTR) can be stimulated by the addition of forskolin leading to an activation of the adenylate cyclase and elevating intracellular cyclic adenosine monophosphate (cAMP) levels [33]. This Cl^-^ secretion leads to an increase of the I_sc_ in Ussing chamber experiments performed in this study. The same mechanism has been shown in native porcine intestinal epithelium [29, 34, 35]. *CFTR* expression was further confirmed by qPCR analysis, showing significantly higher expression in 3D organoids compared with native tissue. This further supports the high increase of I_sc_ using the organoid-based 2D monolayer. However, histological analysis showed CFTR expression in a few cells indicating a high transport capacity. Besides Cl^-^ secretion, CFTR plays an important role regarding bacterial growth in the intestines [36–38] as well as composition of epithelium-covering mucus [39]. Decreasing tissue resistance due to the addition of forskolin is mediated by the activation of protein kinase A due to higher cAMP levels leading to a modification of the tight junction barrier as has been shown in several other studies [40–42].

Further Ca^2+^-dependent Cl^-^ secretion [43] resulting in an increase in I_sc_ could be demonstrated in our system by addition of carbachol and potential activation of calcium activated calcium channels [44], which has already been shown for native porcine epithelium [45, 46].

Physiological transport properties induced by different agents investigated in this study are highly comparable to the response of native tissue obtained in earlier studies. This clearly demonstrate the suitability of the present model as an alternative for animal-based studies such as Ussing chamber experiments, which needs freshly obtained material from slaughtered animals. In contrast, our organoid-based system allows the ongoing cultivation of the epithelium without the need of new native material, as it has been reviewed by Seeger [47].

### Incubation with CTX leads to pathophysiological responses

In our experiments, incubation with CTX led to an increase in I_sc_. This was likely the result of the activation of the adenylate cyclase leading to elevated cellular cAMP levels, resulting in an increased Cl^-^ secretion and decreased sodium absorption [48, 49]. In more detail, Cl^-^ secretion is mediated either by phosphorylation of CFTR [50] or by recruitment of CFTR to the apical membrane of the cells [51]. Furthermore, in our setup, incubation with CTX resulted in a decrease of R_t_. This decrease can be explained by the disruption of the epithelial barrier and disturbance of the cellular junctions [52]. Nevertheless, infections with *Vibrio cholerae* do not cause clinically relevant infections in pigs. This probably depends on components, which are solely found in the intestine and especially in the mucus of pigs that allow the binding of the toxin and therefore impede its effects [53]. Moreover, these components, chemically identified as neutral glycosphingolipid, seem to depend on the ABO blood type of the infected organism [54]. Since 1977 [55] an association between blood type and severity of cholera infection was recognized. Recent studies revealed a direct molecular link between blood group and CTX infection [56], but the detailed cellular mechanism still remains unclear. Despite the clinical irrelevance for pigs, CTX was used in this study as a proof of concept, enabling the comparison to earlier studies and models [Hoffmann et al. 2021, under revision at: PLOS ONE].

After treatment with CTX, forskolin was still able to induce an increase in I_sc_. This was not shown in an earlier study using the Caco-2/HT29-MTX [Hoffmann et al. 2021, under revision at: PLOS ONE] and one could speculate that this is impeded by the full activation of the CFTR. However, qPCR reveals high abundance of the transporter and the toxin itself is, as discussed before, supposed to be less harmful using porcine material.

Although this part of the study can be regarded as a proof of concept, mimicking pathophysiological *in vivo* reactions, this enhances the suitability of our model for further pathophysiological relevant questions such as the investigation of effects of other bacterial toxins or living pathogens.

## Conclusion

With the present study we were able to establish a porcine organoid-based model of the small intestine generated from adult stem cells from intestinal crypts forming the investigated organoids. This study further proves the feasibility to use this model for investigating physiological (e.g. glucose transport or Cl^-^ secretion) and pathophysiological responses to CTX as a proof of concept. Expression of tight junction associated proteins, as well as transport characteristics are comparable with data obtained from native porcine epithelium. This highlights the suitability of our organoid-based model to replace the use of native epithelium. Taken together, the present study proposes a model with great potential as an alternative to classically used intestinal tissue samples directly obtained from pigs, implicating a possibility to reduce animal experiments.

## Supporting information

S1 Table Culture medium composition. (DOCX)

S2 Table Primers used for gene expression quantification. (DOCX)

S3 Table Monolayer medium composition. (DOCX)

S4 Table Differentiation medium composition. (DOCX)

S5 Table Composition of the buffer solutions used for Ussing chamber experiments (all chemicals were obtained from Sigma-Aldrich, Darmstadt, Germany and diluted in *aqua destillata*). (DOCX)
