# Supplementary material for: Intestinal organoid-based 2D monolayers mimic physiological and pathophysiological properties of the pig intestine
Source: PLoS One. 2021 Aug 23;16(8):e0256143. doi: 10.1371/journal.pone.0256143 (PMC8382199; doi:10.1371/journal.pone.0256143)
Supplement: S1 Table — (DOCX) [file pone.0256143.s001.docx]

**S1 Table:** Culture medium composition

| **culture medium ingredients** | **manufacturer** | **catalog no.:** |
| --- | --- | --- |
| Advanced DMEM/F12 supplemented with: | Thermo Fisher Scientific, Waltham, USA | 12634010 |
| 2 mM GlutaMax | Thermo Fisher Scientific | 35050061 |
| 50 % (v/v) LWRN-supernatant | Selfmade, according to [1] |  |
| 10 mM HEPES | Biochrom, Berlin, Germany | L 1613 |
| 100 U/ml penicillin 100 µg/ml streptomycin | Thermo Fisher Scientific | 15140122 |
| 800 µl B27 supplement | Miltenyi, Bergisch Gladbach, Germany | 130-097-263 |
| 50 ng/ml recombinant murine EGF | Prepotech, New Jersey, USA | 315-09 |
| 1 mM N-Acetyl-L-cysteine | Sigma-Aldrich, Schnelldorf, Germany | A7250 |
| 10 µM Y27632 | Hölzel, Cologne, Germany | T1725 |
| 500 nM A83-01 | MedChemExpress, New Jersey, USA | HY-10432 |
| 10 µM SB202190 | MedChemExpress | HY-10295 |
| 10 nM Gastrin 1 | MedChemExpress | HY-P1097 |

1. Miyoshi H, Ajima R, Luo CT, Yamaguchi TP, Stappenbeck TS. Wnt5a potentiates TGF-beta signaling to promote colonic crypt regeneration after tissue injury. Science. 2012;338(6103):108-13.
