# Supplementary material for: Intestinal organoid-based 2D monolayers mimic physiological and pathophysiological properties of the pig intestine
Source: PLoS One. 2021 Aug 23;16(8):e0256143. doi: 10.1371/journal.pone.0256143 (PMC8382199; doi:10.1371/journal.pone.0256143)
Supplement: S2 Table — (DOCX) [file pone.0256143.s002.docx]

**S2 Table:** Primers used for gene expression quantification.

| Target gene | **Forward primer** | **Reverse primer** | **RefSeq Accesion no.:** |
| --- | --- | --- | --- |
| ***SLC5A1 (SGLT1)*** | CAGTCGCCATGGACAGTAGC | GTGGAGCACATAGCCCACAG | NM_001164021.1 |
| ***CFTR*** | GGAGCAGGCAAGACATCACT | GCACGCTTTGATGACACTCC | NM_001104950.1 |
| ***MUC2*** | GGCGATGATTTCAAGACGGC | GCGTAGTTGGCACTCTCGAT | XM_021082584.1 |
