# Supplementary material for: Intestinal organoid-based 2D monolayers mimic physiological and pathophysiological properties of the pig intestine
Source: PLoS One. 2021 Aug 23;16(8):e0256143. doi: 10.1371/journal.pone.0256143 (PMC8382199; doi:10.1371/journal.pone.0256143)
Supplement: S4 Table — (DOCX) [file pone.0256143.s004.docx]

**S4 Table:** Differentiation medium composition.

| **differentiation medium ingredients** | **manufacturer** | **catalog no.:** |
| --- | --- | --- |
| Advanced DMEM/F12 supplemented with: | Thermo Fisher Scientific | 12634010 |
| 5 % (v/v) LWRN-supernatant | Selfmade, according to Miyoshi, Ajima [1] |  |
| 20 % (v/v) fetal bovine serum | Biochrom | S0115 |
| 2 mM L-glutamine | Merck | A0283 |
| 100 U/ml penicillin 100 µg/ml streptomycin | Thermo Fisher Scientific | 15140122 |
| 50 ng/ml recombinant murine EGF | Prepotech | 315-09 |
| 5 µM DAPT | MedChemExpress | HY-1307 |

1. Miyoshi H, Ajima R, Luo CT, Yamaguchi TP, Stappenbeck TS. Wnt5a potentiates TGF-beta signaling to promote colonic crypt regeneration after tissue injury. Science. 2012;338(6103):108-13.
