# Supplementary material for: Intestinal organoid-based 2D monolayers mimic physiological and pathophysiological properties of the pig intestine
Source: PLoS One. 2021 Aug 23;16(8):e0256143. doi: 10.1371/journal.pone.0256143 (PMC8382199; doi:10.1371/journal.pone.0256143)
Supplement: S5 Table — (DOCX) [file pone.0256143.s005.docx]

**S5 Table:** Composition of the buffer solutions used for Ussing chamber experiments (all chemicals were obtained from Sigma-Aldrich, Darmstadt, Germany and diluted in *aqua destillata*).

|  | **Mucosal buffer [mM]** | **Serosal buffer [mM]** | **CAS no.:** |
| --- | --- | --- | --- |
| **NaCl** | 119.6 | 119.6 | S7653 |
| **KCl** | 5.4 | 5.4 | P9333 |
| **HCl** | 0.4 | 0.4 | H1758 |
| **MgCl_2_ * 6 H_2_O** | 1.2 | 1.2 | M2670 |
| **CaCl_2_ * 2 H_2_O** | 1.2 | 1.2 | C7902 |
| **NaHCO_3_** | 15.0 | 15.0 | S5761 |
| **Na_2_HPO_4_** | 1.2 | 1.2 | S9763 |
| **NaH_2_PO_4_** | 0.3 | 0.3 | S0751 |
| **Mannitol** | 20.0 | 10.0 | M4125 |
| **Glucose** | - | 10.0 | G8270 |
| **Indometacin** | 0.01 | 0.01 | I7378 |
